# Checkpoint blocker induced autoimmunity as an indicator for tumour efficacy in melanoma

**DOI:** 10.1038/s41416-021-01390-1

**Published:** 2021-10-25

**Authors:** Jessica C. Hassel

**Affiliations:** Section of DermatoOncology, Department of Dermatology and National Center for Tumor Diseases, University Hospital Heidelberg, Heidelberg, Germany

## Abstract

Immune checkpoint inhibitors (ICI) have improved survival of patients with metastatic melanoma but can induce autoimmunologic side effects. Ye et al. report a retrospective analysis that further supports the finding that these are biomarkers for patients’ clinical benefit. Thereby, patients with immune-related adverse events show a differential gene expression in chemokine-mediated signalling.

## Main

Immune checkpoint blockers (ICB) have substantially changed clinical outcome of metastasised melanoma. Whereas almost all patients with advanced disease eventually died from the melanoma in the era of chemotherapy now in about one third of patients the immune system can be reactivated to control metastatic disease long-term.^1^ ICB thereby inhibit—as their name indicates—so called immune checkpoints such as CTLA4 (cytotoxic T-lymphocyte-associated protein 4) and PD-1 (programmed cell death protein 1) that are expressed to control T cell activation and limit immune response. However, immune activation by ICB is unspecific and enhances activity of T cell clones that have already formed. Hence, not only tumour responses can be induced but autoimmunity triggered also. ICB are known to induce autoimmune side effects so called immune-related adverse events (irAEs) in many organs with skin, gastrointestinal tract, liver and endocrine glands as the most frequent ones.^2^ It still is a matter of debate if the development of irAEs indicates an effective immune activation by ICB and correlates with clinical outcome of the melanoma.^3^ Writing in the *British Journal of Cancer*, Weiyu Ye et al.^4^ report a retrospective study of patients with metastatic melanoma receiving single agent anti-PD-1 or combination therapy with the anti-CTLA-4 antibody ipilimumab. In a first cohort of 144 patients 44% received at least one cycle of ipilimumab plus nivolumab and 56% anti-PD-1 monotherapy. 65% of patients developed irAEs, 58% early within 12 weeks of treatment start and 30% with grade 3/4 severity. Early irAEs consisted of cutaneous irAEs, colitis and hepatitis as the most frequent, typical late irAEs were gastritis and pneumonitis. To prevent a guarantee-time bias only early irAEs were considered in a statistical landmark analysis excluding patients who died within 12 weeks after treatment start. Here, patients with early irAEs revealed a significantly longer overall survival (OS) with a highly reduced risk for death (HR 0.29, 95% CI 0.15–0.58, *p* = 0.0004). This was confirmed in an independent cohort of 211 patients with similar baseline characteristics. In multivariable analysis besides early irAEs monocyte and neutrophil count were associated with OS. In the peripheral blood of pre-treatment samples in patients receiving anti-PD-1 monotherapy 35 transcripts were differentially expressed between the ones developing irAE compared to patients without. These consisted of genes involved in anti-microbial responses, phagocytosis and complement activation. Including on-treatment samples taken 3 weeks after treatment initiation patients with irAEs—independent of treatment type—showed differential gene expression in chemokine-mediated signalling, extracellular matrix organisation and platelet degranulation. Interestingly, increased expression of the interleukin-8 (IL-8) chemokine receptor CXCR1 and to a lesser extent CXCR2 was associated with reduced development of irAEs in pre- and on-treatment samples. Unfortunately, the authors did not correlate the expression with clinical outcome but measured IL-8 serum levels in a subset of patients and found a correlation of high CXCR1 expression and IL-8 in serum. In a recent study it was shown that high IL-8 serum levels were associated with increased intratumoural neutrophils and a reduced clinical benefit to ICB.^5^ In line with this, elevated neutrophils as well as elevated monocytes in the peripheral blood were associated with a shorter OS in several retrospective analyses.^3^

An association of autoimmune side effects with immune response against the tumour seems logical as immune checkpoint blockers lead to an unspecific antigen-independent activation of existing T cell clones. Thereby, the irAE might be induced by tumour specific T cell clones as shown for vitiligo and myocarditis or by different ones.^3, 6^ In vitiligo CD-8+ T cells react against melanocyte differentiation antigens and thereby hit not only the melanoma but normal melanocytes in the skin. Vitiligo was one of the first irAE that had been shown to correlate with clinical outcome in melanoma patients. However, for other irAEs the correlation is less clear with hints for ir-hypophysitis and arthralgia to be associated with longer OS.^3^ Different results from different retrospective analyses might therefore be based partly on patient and irAE composition in generally small patient cohorts. In a meta-analysis of 40 studies with more than 8000 patients, overall response rate, progression free survival (PFS) and OS was significantly better in patients developing irAEs.^7^ Here, preferentially patients with irAEs in skin, endocrine organs or gastrointestinal tract were found to obtain clinical benefit. In most analyses, irAEs of any grade were correlated with response and survival. Three studies illustrated the relationship between severity of irAEs and efficacy and found a longer OS for patients with low-grade irAE compared to the high-grade irAE group.^7^ The reason for this most likely is the immunosuppressive treatment that is needed for irAE management. In a cohort of 169 patients with metastasised melanoma it was shown that high-dose steroid treatment of patients suffering from severe irAE impacts the clinical benefit of ICB therapy demonstrated in a poorer post-irAE PFS.^8^

In conclusion, more and more data support that the development of irAEs indicates treatment efficacy. However, it is important to note that about one third of patients alive at 1 year responded to treatment in the absence of any irAE.^4^ In addition, patients with uveal melanoma rarely benefit from ICB treatment but develop irAEs to a similar extent.^9^ irAEs therefore are only part of the story.

## Data Availability

By review.
